# Value of microbial metabolites in blood serum as criteria for bacterial load in the pathogenesis of hemodynamic disorders in critically ill patients

**DOI:** 10.1186/cc13398

**Published:** 2014-03-17

**Authors:** Y Sarshor, N Beloborodova, V Moroz, A Osipov, A Bedova, E Chernevskaya, M Getsina

**Affiliations:** 1 Negovsky Research Institute of General Reanimatology, Moscow, Russia

## Introduction

Hemodynamic disorders in critically ill patients are often connected with bacterial load. Bacterial load is usually associated with bacteremia, LPS, high level of IL-6, PCT and also with aromatic microbial metabolites [1-3], and so forth. In our opinion, microbial metabolites can participate in hemodynamic disorders in critically ill patients, particularly due to their influence on NO production [4] and intestinal permeability.

## Methods

In a prospective study we observed critically ill patients on the day of admission to a polyvalent ICU, severe cardiac pathology was excluded. The level of phenylpropionic, phenyllactic, p-OH- phenyllactic, p-OH-phenylacetic acids and total phenylcarboxylic acids (PhCAs) were measured in blood serum using gas chromatography (GC-FID). The level of PCT and NT-proBNP were measured using Elecsys 2010. Comparison between patients with hypotension (on vasopressor support) (group A) and without (group B) was performed.

## Results

We studied 50 ICU patients with different diseases: pneumonia (*n *= 15), severe kidney failure (*n *= 13), abdominal surgical pathology (*n *= 10), alcoholic cirrhosis (*n *= 5), soft-tissue infection (*n *= 7). In group A (24/50) the median of PhCAs was 17.8 (IR 11.4 to 30.0) μmol/l, and in group B (26/50) it was 7.2 (IR 3.7 to 13.2) μmol/l, *P *= 0.003 (t test). In group A, all patients (with or without documented infections) had symptoms of infection manifestation [5], 20/24 (83.3%) of them died. In group B, the symptoms of infection manifestation were revealed in 12/26 (46%) cases, and the mortality was significantly lower, 3/26 (11.5%) (*P *< 0.05). General mortality was 23/50 (46%). The profile of PhCAs differed in groups A versus B.

## Conclusion

The total level of PhCAs in critically ill patients with hypotension was considerably higher than in hemodynamically stable patients. The participation of microbial factor in pathogenesis of hemodynamic disorders in the presence of systemic inflammation may be validated with the load of microbial metabolites (PhCAs).

## Acknowledgements

This work was supported by the Russian Foundation for Basic Research (project number 13-04-01758/13).
